# From Ionic Agitation to Precision Neuroablation: A Bioengineering Perspective on the Mechanisms, Modalities, and Clinical Applications of Radiofrequency Technology in Pain Management

**DOI:** 10.3390/bioengineering13101102

**Published:** 2026-09-23

**Authors:** Eric E. Poole, Charles A. Odonkor, Eric T. Nguyen, David W. Lee, Mustafa R. Dodurgali, Emily Draeger, Alaa Abd-Elsayed

**Affiliations:** 1 Department of Physical Medicine and Rehabilitation, Charles R. Drew University of Medicine and Science, 1731 E. 120th St., Los Angeles, CA 90059, USA; ericpoole@cdrewu.edu (E.E.P.); ericnguyen@cdrewu.edu (E.T.N.); 2 Department of Orthopedics and Rehabilitation, Division of Physiatry, Interventional Pain Medicine, Yale New Haven Hospital, 20 York Street, New Haven, CT 06510, USA; mustafa.dodurgali@yale.edu; 3 Department of Therapeutic Radiology, Yale University School of Medicine, 333 Cedar Street, New Haven, CT 06510, USA; emily.draeger@yale.edu; 4 Department of Physical Medicine and Rehabilitation, University of California, Irvine, 101 The City Drive South, Orange, CA 92868, USA; lee.davidw@gmail.com; 5 Department of Anesthesiology, University of Wisconsin, Madison, 600 Highland Avenue, Madison, WI 53792, USA; abdelsayed@wisc.edu

**Keywords:** radiofrequency ablation, neuromodulation, basivertebral nerve, spine pain, peripheral nerves, electroporation, bioheat modeling, pain interventions

## Abstract

Radiofrequency ablation (RFA) is a foundational interventional technology in pain management. Despite the diversity of available systems, conventional thermal, internally cooled, bipolar, and pulsed radiofrequency (PRF) all share one biophysical mechanism: high-frequency alternating current (AC) drives tissue ions to oscillate, generating frictional heat within the tissue surrounding the electrode. This localized heating produces predictable patterns of coagulative necrosis shaped by tissue electrical conductivity, electrode geometry, power delivery, and perfusion-related heat dissipation. Classical and contemporary bioheat models, including the Pennes bioheat equation, describe how these variables determine lesion size and morphology. Advances in device engineering have reshaped lesion characteristics through internally cooled probes, bipolar strip-lesioning systems, and pulsed platforms that modulate neural activity while preserving structural integrity. These innovations broaden therapeutic options by enabling targeting of anatomically variable structures, such as the sacral lateral branches, and intraosseous targets, including the basivertebral nerve (BVN). Emerging technologies—high-voltage PRF, hybrid pulsed–thermal systems, and electroporation-based pulsed-field ablation—reflect a growing emphasis on tissue selectivity, energy efficiency, and safety. This narrative review traces the historical development of radiofrequency neurotomy, examines the biophysical principles governing lesion formation (including the Pennes bioheat equation and the roles of current density, impedance, and perfusion), and details the engineering distinctions among modalities. Clinical applications across spinal, joint, and peripheral nerve targets are presented to illustrate how these physical and engineering differences translate into procedural decision-making, and emerging modalities such as high-intensity focused ultrasound (HIFU) and pulsed-field ablation are discussed in the context of the biophysical principles they extend or depart from.

## 1. Introduction

All radiofrequency systems used in interventional pain management share the same fundamental mechanism: high-frequency alternating current (AC), typically 300–500 kHz, causes tissue ions to oscillate and generate frictional heat within the surrounding tissue rather than at the electrode itself. This principle, in which the tissue itself becomes the heat source, distinguishes radiofrequency ablation (RFA) from direct thermocoagulation and electrocautery, and is examined in detail in Section 3 [1,2,3,4].

Although this biophysical mechanism is shared, the clinical behavior of radiofrequency systems varies substantially because each modality shapes the electric field, controls temperature, and determines lesion geometry differently. Conventional thermal ablation maintains tissue at 80–90 °C, producing elliptical lesions whose growth is constrained by rising impedance as tissue desiccates [5,6]. Internally cooled electrodes prevent surface charring, allowing higher power deposition and producing larger, more spherical lesions, an advantage in anatomically variable regions such as the sacral lateral branches [4,6,7,8]. Bipolar configurations concentrate current between two active electrodes, generating elongated strip lesions that improve the probability of capturing nerves with variable trajectories [7,9,10,11]. Pulsed radiofrequency (PRF) systems deliver high-voltage bursts at temperatures below the threshold for coagulative necrosis, producing neuromodulatory effects through electroporation and altered membrane potential rather than thermal neurodestruction [9,10,11,12,13,14].

Understanding these engineering distinctions is essential for rational modality selection. The choice between conventional, cooled, bipolar, and pulsed systems is not arbitrary; it is determined by the anatomy of the target nerve, the tissue environment, and the clinical goal, whether neurodestruction or neuromodulation [1,2,3,4,15]. Yet the underlying physical principles and their clinical implications are not always well integrated in contemporary reviews or practice. Throughout this review, conventional monopolar, internally cooled, and bipolar radiofrequency are treated together as thermal modalities that share a coagulative mechanism but differ in lesion geometry. PRF is treated as a distinct nonthermal neuromodulatory technique; PFA is treated as a separate nonthermal, electroporation-based modality at an early stage of clinical translation for pain indications [12,13,14].

This review addresses that gap. Beginning with the historical development of radiofrequency neurotomy, it examines the biophysical principles governing lesion formation, details the engineering differences among modalities, and discusses how patient-specific tissue factors modify lesion behavior. Clinical applications across spinal, joint, and peripheral nerve targets are presented not as standalone reviews but as illustrations of how physics and engineering translate to procedural decision-making. Emerging technologies, including hybrid pulsed–thermal systems and pulsed-field ablation, are discussed in the context of the biophysical principles they extend or depart from. In doing so, this review complements recent overviews of radiofrequency ablation [16,17,18] by explicitly linking the biophysics of lesion formation to device engineering and to target-specific modality selection, rather than cataloging modalities in isolation.

Scope and literature approach. This is a narrative, non-systematic review. We searched PubMed/MEDLINE and Scopus through 2025 for English-language work on radiofrequency and energy-based neuroablation in pain medicine, prioritizing randomized trials, society guidelines, mechanistic and bioengineering studies, and ex vivo lesion-characterization data, and we hand-searched the reference lists of recent reviews [16,17,18]. Sources were selected for mechanistic relevance and representativeness rather than for exhaustive coverage, and no formal systematic review or meta-analytic criteria were applied. This approach fits the review’s aim of connecting biophysics, device engineering, and clinical practice; its principal limitation, addressed again below, is the selection bias inherent to narrative synthesis.

## 2. Historical Development of Radiofrequency Neurotomy

The application of radiofrequency energy to neural tissue has evolved over nearly a century, progressing from rudimentary thermocoagulation to a diverse family of modalities, including conventional thermal, pulsed, cooled, and bipolar systems. Understanding this trajectory clarifies how each engineering advance arose in response to specific clinical limitations and why the field continues to expand.

The earliest known use of radiofrequency current for neural ablation dates to the early 1930s, when German neurosurgeon, Martin Kirschner applied thermocoagulation to the Gasserian ganglion to treat trigeminal neuralgia [5]. Kirschner’s work established the foundational principle that a continuous radiofrequency current could create a focal thermal lesion within a neural pathway, thereby interrupting nociceptive transmission [4,5]. Although the technique was effective, it remained limited by the absence of commercially available generators and standardized electrodes. Two decades later, in the 1950s, Sidney Aronow and Bernard Cosman developed the first commercially available radiofrequency systems, which made controlled lesion generation reproducible and accessible to a broader surgical community [4,5].

The transition from cranial to spinal applications occurred through a series of innovations in the 1960s and 1970s. W.S. Rees introduced “percutaneous rhizolysis” in 1960, and C. Norman Shealy became the first to apply radiofrequency electrodes specifically for facet joint denervation in 1971 [5]. These early spinal procedures were refined through collaborations with Cosman medical-device engineers during the 1970s, resulting in purpose-built electrode systems, including the Shealy Rhizolysis Kit, the Ray Rhizotomy Electrode, and the Sluijter-Mehta Kit [5]. A pivotal conceptual advance came in 1980, when Nikolai Bogduk and Donlin Long formalized “percutaneous lumbar medial branch neurotomy,” a technique that largely replaced open surgical neurotomy and established the anatomical and procedural framework that remains the basis for modern facet joint RFA [5]. Bogduk continued to contribute significantly to the validation, refinement, and broader acceptance of the technique over the following decades [5].

Despite the clinical success of conventional thermal ablation, a persistent concern remained: the risk of motor deficit when treating targets near mixed sensorimotor nerves. This limitation motivated the development of PRF, which was introduced in Austria in 1995 by Meno Sluijter, Eric Cosman, William Rittman, and Maarten van Kleef [4,5]. By delivering high-voltage bursts separated by prolonged silent intervals, PRF maintained tissue temperatures below the threshold for coagulative necrosis while still exerting measurable biological effects on neural tissue [2,4]. The first article describing the effects of PRF on the dorsal root ganglion was published in 1998, and the technique rapidly gained interest as a non-neurodestructive alternative for radicular pain and peripheral neuralgias [2,4,5].

A separate engineering challenge, the limited lesion size achievable with conventional monopolar probes, drove the development of cooled radiofrequency technology. The internally cooled electrode concept was first demonstrated by Torben Lorentzen in 1996 for hepatic tumor ablation [19]. By circulating fluid through the cannula, cooled probes prevent tissue charring and desiccation at the electrode surface, thereby allowing higher power deposition and producing larger, more spherical lesions than conventional devices of equivalent gauge [4,19]. This technology was initially adopted in cardiac electrophysiology and oncologic ablation before being adapted for pain management applications. Since 2010, a growing body of evidence has supported the use of cooled radiofrequency for chronic knee, hip, sacroiliac joint, and lumbar facet pain, where its larger lesion volumes are advantageous (Section 5.2) [4,6,19,20].

The most recent chapter in this history includes the introduction of bipolar configurations, intraosseous approaches such as basivertebral nerve (BVN) ablation, and emerging nonthermal platforms, including pulsed-field ablation. Each of these innovations represents a response to specific anatomical or physiological challenges that earlier modalities could not fully address. Several recent reviews have surveyed the clinical landscape of RFA in pain management, including overviews of modality selection and technique [16,17,18].

The present review builds on this body of work by focusing specifically on the biophysical mechanisms that distinguish each modality and by examining how engineering differences in electrical field behavior, temperature control, and lesion geometry translate to clinical decision-making. Taken together, the historical arc of radiofrequency neurotomy reflects a consistent pattern: clinical observation identifies a limitation, engineering innovation produces a new modality, and subsequent clinical investigation defines its optimal application. The sections that follow examine the biophysical principles, engineering distinctions, and clinical evidence that have emerged from this iterative process.

## 3. Physical Principles of Radiofrequency Energy

RFA relies on the delivery of oscillating electromagnetic energy in the radio-wave band of the electromagnetic spectrum to heat tissue, applied to the treatment site through an electrode. The frequencies used for RFA are low enough that cell membranes cannot depolarize and generate action potentials, yet high enough to drive ion oscillations (300–500 kHz). As the electric field alternates between positive and negative potential, ions shift back and forth within the tissue, creating frictional heat. This frictional (resistive) heating raises the temperature within the tissue itself rather than at the electrode surface, although the surrounding tissue passively heats the electrode as tissue temperature rises [2,6,8,21].

Radiofrequency energy deposition within the tissue depends on several factors, including current density, tissue impedance (the opposition to AC, governed chiefly by tissue electrical conductivity), exposure time, and electrode size [21,22,23]. Because of the interplay among these factors, the size and shape of the ablation zone are difficult to predict. In 1948, Harry H. Pennes used a thermocouple and galvanometer to measure temperature changes in the human forearm and derived an equation relating blood and tissue temperatures [24]. This equation, known as the Pennes bioheat equation, remains widely used to describe temperature variations within human tissue and is given by:

$$\rho c\frac{\partial T}{\partial t}=\nabla \cdot \left(k\nabla T\right)-\rho_{b}c_{b}\omega_{b}\left(T-T_{b}\right)+Q_{m}+Q_{p}$$

where ρ is the tissue density (kg/m^3^), c is the specific heat capacity of the tissue (J/kg K), k is the thermal conductivity of the tissue (W/m K), $\rho_{b}$ is the density of blood (generally taken as 1050 kg/m^3^), $c_{b}$ is the specific heat of blood (generally taken as 3617 J/kg/K), $\omega_{b}$ is the blood perfusion rate (1/s), $Q_{p}$ is the volumetric heating due to the electromagnetic source (W/m^3^), $Q_{m}$ is the metabolic heat generation in the tissue (W/m^3^), $T_{b}$ is the core blood temperature (generally taken as 37 °C), and T is the tissue temperature. $Q_{p}$ is determined based on the electric field (*E* = V/m) and current density (*J* = A/m^2^) generated within the tissue: $Q_{p}=J\cdot E$ [25]. An illustration of the Pennes bioheat model is provided in Figure 1.

One limitation of the Pennes bioheat equation is its simplified treatment of blood perfusion. It represents perfusion as a spatially uniform heat-transfer and therefore does not account for vessel size, flow velocity, or flow direction. Consequently, extrapolation of results to perfused, innervated human tissue is uncertain. Despite this simplification, the Pennes equation has been used extensively and shows good agreement with experimental data [26]. Other model-based approaches have also been applied to RFA modeling, including the porous-medium bioheat transfer model [27] and the Wulff, Klinger, Chen–Holmes, and Weinbaum–Jiji models [28].

Within the tissue, current density is highest at the surface of the active electrode and decreases with distance, producing the characteristic ellipsoidal thermal gradient of conventional ablation (Figure 2a). Current density depends on electrode size and applied current; for an applied current of 500 mA and an electric-field intensity of 300 V/m, increasing the probe surface area from 10 mm^2^ to 30 mm^2^ enlarges the ablation zone and shortens the time to reach a given temperature, as shown in Figure 2a,b.

To illustrate these relationships qualitatively, the bioheat model can be evaluated with representative parameters. Using a thermal conductivity *k* ≈ 0.5 W·m^−1^·K^−1^, tissue density *ρ* ≈ 1050 kg·m^−3^, specific heat *c* ≈ 3600 J·kg^−1^·K^−1^, and electrical conductivity *σ* ≈ 0.3 S·m^−1^, the volumetric source term *Q* = *σ*|*E*|^2^ at the field intensities encountered near the active tip (of the order of 10^2^–10^3^ V·m^−1^) deposits energy quickly enough to drive local tissue above the 60 °C coagulation threshold within tens of seconds, qualitatively consistent with the time-to-temperature curves in Figure 2b [24,25]. Because *Q* scales with the square of the electric field and the field decays steeply with distance, the 60 °C isotherm that defines the lesion edge remains within roughly 1–3 mm of a small monopolar tip, an order-of-magnitude that is broadly compatible with the 169–360 mm^3^ ex vivo lesion volumes reported for 16- to 20-gauge probes [8]. The model therefore identifies two practical levers for enlarging the lesion: increasing the active-tip surface area, which raises the heated volume and shortens the time to a given temperature (Figure 2, 10 vs. 30 mm^2^), and internal cooling, which permits higher applied voltage and current before charring and thereby shifts the same physics outward, toward the ~595 mm^3^ volumes of cooled probes [8,29]. The perfusion term, *ρ_b_c_b_ω_b_* (*T* − *T_b_*), opposes lesion growth: in well-perfused tissue, the predicted lesion radius can fall substantially, which is why computational models report lesion-size variation exceeding 100% with local blood flow [22] and why bony, poorly perfused targets such as the BVN retain heat and lesion predictably [27].

These calculations are intended to be illustrative rather than predictive. They rely on generic, tissue-averaged literature parameters and on the simplifying assumptions of the Pennes formulation, most notably a single uniform perfusion term that ignores discrete vessel size, flow velocity, and flow direction, so the resulting values should be read as first-order approximations that convey the qualitative dependencies among current density, tip geometry, impedance, and perfusion, not as patient-specific predictions of lesion dimensions (Section 10) [26,27]. The apparent correspondence with ex vivo lesion volumes reflects a plausibility check rather than a formal validation, since the model was not independently fit to, or verified against, those measurements. The limitations of extrapolating ex vivo and computational lesion estimates to perfused, innervated human tissue are discussed further in Section 10 (Controversies, Limitations, and Evidence Gaps). With these caveats, the model helps rationalize, rather than establish, the modality and target choices developed in the sections that follow.

Another factor that modulates the degree of heating is tissue impedance, which is determined by tissue electrical conductivity. Differences in fluid composition among tissues lead to varying electrical conductivity. As fluid is removed from the tissue during RFA, impedance increases, thereby limiting further energy transfer. Most lesion expansion, therefore, occurs in the early portion of an ablation cycle before impedance rises significantly. Highly perfused regions dissipate heat rapidly and tend to form smaller lesions, whereas bone and low-conductivity tissues trap heat. These principles are evident in spine procedures such as medial branch neurotomy, in which nerves course along osseous structures that alter thermal flux [1,9,23,29]. Impedance can also transiently rise from gas formation between the electrode and tissue as fluid evaporates during treatment [21].

Treatment time depends on the radiofrequency system used. In general, energy is applied until the target temperature is reached, then the treatment is switched on and off to maintain that temperature, with treatment typically lasting a few minutes. Temperatures sustained above approximately 60 °C reliably cause protein denaturation and coagulative necrosis, which forms the basis for conventional lesion targets [29]. These principles provide the foundation for evaluating and optimizing modern radiofrequency techniques.

## 4. Mechanisms of Tissue Interaction

The biological response to RFA depends on whether the applied energy produces thermal neurodestruction, nonthermal neuromodulation, or both. The distinction depends primarily on temperature and the pattern of electric-field exposure [1,4,29,30,31,32]. The various RFA techniques, procedural parameters, and expected clinical outcomes across primary anatomical targets are summarized in Table 1.

Thermal mechanisms dominate at temperatures above approximately 60 °C. At this threshold, proteins denature, intracellular enzymes fail, and cell membranes lose structural integrity. Conventional thermal RFA deliberately maintains these temperatures for sixty to ninety seconds to achieve consistent neural injury [1,4]. Following exposure, axons undergo fragmentation and demyelination, after which Wallerian degeneration removes distal neural elements over several weeks. Because DRG cell bodies persist, axonal regrowth may occur, accompanied by remyelination, typically within 3–6 months. These mechanisms underlie the typical duration of sensory denervation and clinical benefit achieved with lumbar facet medial branch neurotomy and other thermal procedures [3,32].

PRF avoids thermal neurodestruction by maintaining tissue temperatures at or below approximately 42 °C while delivering high-voltage bursts separated by prolonged silent and refractory intervals [32]. By keeping temperatures subthreshold, PRF avoids heat-related complications such as neuritis, motor dysfunction, or deafferentation pain. Energy is delivered as high-voltage bursts, typically two pulses per second at 500 kHz, with each pulse lasting 20 milliseconds over 120 s. The low average temperature prevents thermal neurodestruction, yet the electric field exerts measurable biological effects. Experimental work demonstrates reversible ultrastructural changes in axons, including alterations in mitochondrial membranes and disruption of microtubules and microfilaments. Preclinical work suggests these effects may be more pronounced in small, unmyelinated C-fibers than in larger myelinated A-delta and A-beta fibers, though fiber selectivity has not been confirmed in clinical settings. Because PRF provides neuromodulation rather than complete sensory denervation, it is most useful for pain syndromes in which neural injury already exists or where motor preservation is essential. Although the temperature remains subthreshold for protein coagulation, the electric fields alter neuronal physiology through mechanisms demonstrated in both animal and in vitro models [12,13,14]. Experimental work suggests that PRF may inhibit axonal transport, reduce pro-inflammatory neuropeptide expression, and increase membrane permeability; these events have been attributed to transient reversible electroporation and are thought to result in neuromodulation that can relieve pain while preserving neural structure. These mechanisms are derived from animal and in vitro models and have not been established in humans [29,32].

The electric-field distribution also influences tissue response. According to computer modeling studies, field strength and orientation affect the extent of electroporation, particularly in bipolar configurations, where fields may overlap and intensify between the electrodes [9]. Neural architecture also matters; small, lightly myelinated fibers are more susceptible to thermal injury, while mixed motor and sensory nerves may be better suited to pulsed techniques [22,33]. Tissue composition, including fat content and perfusion, further modulates lesion formation [23,29,32,34]. These mechanisms and variations contribute to the complexity of clinical outcomes and reinforce the importance of optimizing modality selection based on anatomy and clinical goals.

### Neural Regeneration and Lesion Durability

Lesion durability is limited by peripheral nerve regeneration. Although regrowth is beneficial after traumatic injury, it presents a challenge in pain management because successful regeneration after neuroablation can lead to symptom recurrence. Repair begins rapidly and proceeds through three principal mechanisms: remyelination of surviving fibers, collateral sprouting from intact axons, and true axonal regeneration [35,36,37,38]. Collateral sprouting predominates when only a minority of axons are damaged. In contrast, complete regeneration dominates when most fibers are injured, and the likelihood of successful regeneration depends heavily on the distance between the proximal stump and the injury site.

Following injury, Schwann cells create a growth-permissive environment and retrograde signaling shifts the neuron toward a regenerative phenotype [35]. Importantly, regeneration is often imprecise; incomplete reinnervation, misdirected axonal targeting, or aberrant motor–sensory reconnection can occur, which helps explain why pain may recur after neuroablation and why lesion durability remains a key consideration when selecting modality, lesion parameters, and target anatomy.

## 5. Radiofrequency Modalities

Radiofrequency systems have evolved to address limitations in lesion size, tissue variability, and safety associated with conventional thermal ablation. Although all modalities share the same biophysical mechanism (Section 3), each shapes the electric field, controls temperature, and determines lesion geometry through distinct engineering strategies [15]. The following subsections detail how each modality works, what makes it unique, and where it is best applied. The principal mechanistic distinctions are summarized in Table 1, and representative quantitative parameters and clinical outcomes are compared in Table 2. Lastly, a visual overview of the distinct electrode configurations, temperature profiles, and expected lesion geometries associated with conventional RFA, cooled RFA, bipolar RFA, and PRF is provided in Figure 3.

### 5.1. Conventional Thermal (Monopolar) RFA

Conventional thermal RFA uses a single active electrode through which continuous AC flows to a dispersive grounding pad affixed to the patient’s skin. The small cross-sectional area of the electrode tip results in a high current density relative to the large grounding pad, thereby concentrating resistive heating in the tissue immediately surrounding the electrode [2,6]. Continuous current delivery maintains tissue at a set temperature, typically 80–90 °C, for 60–90 s [1,3,4].

The resulting lesion is elliptical or teardrop-shaped, with its maximal diameter near the active tip. Current density is highest at the electrode surface. It decays steeply with distance, so resistive heating is confined to approximately 1–2 mm from the electrode, with deeper injury occurring via passive conductive heat transfer [2,6]. Lesion expansion slows as tissue desiccates and impedance rises, a self-limiting process that constrains lesion volume; approximately 87% of the maximal lesion area is achieved within the first 60 s after the set temperature is reached, although continued application reduces lesion-size variability [45]. Ex vivo studies report lesion volumes ranging from 169 mm^3^ (20-gauge) to 360 mm^3^ (16-gauge) at 80 °C for 90 s [8].

Conventional thermal RFA remains the most widely used modality and is the reference standard for lumbar medial branch neurotomy, supported by high-quality evidence and multiple society guidelines [1,3,4]. Its principal advantages are simplicity, predictability, and extensive clinical validation; its principal limitation is the constrained lesion volume imposed by impedance rise, which can result in incomplete nerve capture when target nerves are anatomically variable or positioned beyond the effective lesion radius.

### 5.2. Cooled RFA

Internally cooled electrodes were developed to overcome the lesion-size limitations of conventional thermal systems. A closed loop perfusate circulates within the cannula, acting as a heat sink that removes heat from the tissue closest to the electrode surface [8,29,30]. This cooling prevents charring, desiccation, and steam formation at the electrode–tissue interface, processes that would otherwise raise impedance and limit further energy delivery. By preventing this impedance rise, cooled electrodes permit higher voltages and greater current deposition into deeper tissue, not because cooling itself “enhances power delivery,” but because it removes the thermal barrier that would otherwise force the system to reduce output [6,8].

The electrode surface temperature is maintained at approximately 60 °C for 150 s, while internal tissue temperatures exceed this level at depth, resulting in larger, more spherical lesions than those produced by conventional probes of comparable gauge [8,29]. Ex vivo studies demonstrate that a 17-gauge cooled probe with a 4 mm active tip produces a mean lesion volume of 595 mm^3^, significantly larger than the 169–360 mm^3^ range produced by 16- to 20-gauge monopolar probes [8]. Significant tissue ablation also occurs distal to the cannula tip with cooled electrodes, whereas conventional probes produce minimal lesioning beyond the tip [30].

Cooled RFA was developed for tumor ablation in the 1990s, adapted for cardiac electrophysiology, and later introduced into pain management after 2010 [19]. Its larger, more uniform lesion geometry is particularly valuable for anatomically variable targets such as the sacral lateral branches and for joints such as the knee, where conventional lesion volumes may be insufficient to capture nerves with unpredictable trajectories [4,8,39,40,46,47]. Limitations include the requirement for specialized equipment and larger-gauge introducers.

### 5.3. Bipolar RFA

Bipolar RFA replaces the single electrode-to-grounding-pad circuit with two active electrodes placed in proximity. Current flows between the two electrodes rather than through the patient’s body to a dispersive pad, concentrating the electric field in the interelectrode tissue and producing an elongated strip lesion that bridges the distance between the probes [7,9,10,11].

Lesion confluence, the formation of a continuous thermal corridor between the electrodes, depends critically on interprobe distance (IPD), electrode gauge, and tissue environment. An ex vivo study by Wondra et al. systematically evaluated these parameters and found that lesion confluence was maintained at IPDs less than 12 mm for 18-gauge probes, less than 10 mm for 20-gauge probes, and less than 8 mm for 22-gauge probes [7]. Beyond these distances, confluence was lost and the two electrodes produced separate, non-contiguous lesions. Notably, all three gauges demonstrated a sharp decline in lesion midpoint area as IPD increased from 8 to 10 mm, suggesting that 8 mm represents a practical threshold for reliable confluence across probe sizes [7].

The strip-lesion geometry of bipolar RFA improves the probability of capturing nerves with variable or unpredictable trajectories. This configuration is particularly useful for the DRG, sacroiliac complex, and intraosseous targets such as the BVN, where the target spans a broader territory than a single monopolar lesion can cover [7,9,10,11]. Computational models confirm that electric-field synergism occurs between appropriately spaced bipolar electrodes, with field convergence enhancing heating in the interelectrode tissue [9]. Limitations include more complex probe positioning and the risk of failed nerve capture if spacing exceeds the confluence threshold.

### 5.4. Pulsed Radiofrequency

PRF occupies a distinct conceptual space because its goal is neuromodulation rather than neurodestruction. Instead of continuous current delivery, pulsed systems deliver high-voltage bursts, typically 20-millisecond pulses at 2 Hz and 500 kHz, separated by prolonged silent intervals that allow heat to dissipate [12,13,14,32]. Tissue temperature is maintained at or below approximately 42 °C, well below the threshold for coagulative necrosis.

Because no thermal lesion is produced, the biological effects of PRF are mediated by the electric field itself rather than by heat. In vitro evidence indicates that PRF can induce electroporation-mediated calcium influx at field strengths above approximately 330–480 V/cm, with cumulative effects increasing over a train of bursts [14]. Preclinical studies report that PRF preferentially suppresses C-fiber-mediated spinal nociceptive transmission while largely sparing A-fiber conduction; these findings derive from animal and in vitro models and have not yet been validated in humans [12,34]. Additional proposed mechanisms, characterized largely in animal and in vitro models, include inhibition of axonal transport in small DRG neurons, suppression of calcitonin gene-related peptide (CGRP) and substance P, and downregulation of pro-inflammatory cytokines with attenuation of microglial signaling; these anti-inflammatory effects have not been established as clinical mechanisms in humans [12,13,34].

Pulse-dose radiofrequency is a technical refinement in which the amplitude and width of each consecutive pulse are held constant, ensuring a consistent delivered dose between patients regardless of tissue variability [41].

PRF is preferred for mixed nerves or targets where motor preservation is essential, including the DRG, occipital nerve, and various peripheral neuralgias [10,11,12,13,14,32,36,48]. Its principal limitation is a shorter duration of clinical benefit compared with thermal ablation, typically 3 to 6 months versus 6 to 12 months, reflecting the non-destructive nature of the intervention [1,32].

### 5.5. Hybrid Pulsed–Thermal Systems

Hybrid systems alternate between pulsed bursts and moderate thermal energy delivery, with temperatures typically reaching 45–60 °C depending on the device. The rationale is to combine the neuromodulatory effects of PRF with partial neurodestruction from mild heating, potentially achieving a longer-lasting benefit than PRF alone while avoiding the complete neural destruction associated with conventional thermal ablation.

Early clinical data suggest benefit in trigeminal neuralgia and other neuropathic disorders, where targeted neurodestruction and neuromodulation may both play therapeutic roles [41,49]. However, preclinical data supporting hybrid platforms remain limited, and no randomized controlled trials have established their superiority over conventional or pulsed systems alone. The mechanisms by which alternating pulsed and thermal exposures interact at the cellular level, and whether the combined effect is additive, synergistic, or simply sequential, have not been formally characterized, an important gap that should be acknowledged when considering these platforms for clinical use.

### 5.6. Pulsed Field Ablation (Irreversible Electroporation)

Pulsed-field ablation (PFA) represents a fundamentally different approach to tissue ablation. Rather than relying on thermal injury, PFA uses high-voltage, ultrashort (microsecond-scale) electrical pulses to create irreversible nanoscale pores in cell membranes, disrupting cellular homeostasis and causing cell death through irreversible electroporation [50]. Temperature remains near physiologic levels throughout the procedure.

The most clinically significant property of PFA is tissue selectivity. Different tissue types have different threshold field strengths for electroporation, so PFA can preferentially ablate one tissue type while sparing adjacent structures. In cardiac electrophysiology, where PFA has been most extensively studied, cardiomyocytes have among the lowest electroporation thresholds of any tissue, allowing myocardial ablation while sparing the esophagus, phrenic nerve, and pulmonary veins [50,51]. The ADVENT trial, a multicenter randomized comparison of PFA with conventional thermal ablation for paroxysmal atrial fibrillation, demonstrated noninferior efficacy with safety advantages, including no cases of esophageal injury, phrenic nerve palsy, or pulmonary vein stenosis [50]. Computational models show that PFA lesions are wider, more symmetrical, and less dependent on blood flow than thermal RFA lesions [22].

Pain management applications of PFA remain investigational. The translational rationale is that the tissue-selective properties observed in cardiac ablation may offer safety advantages for neural ablation in anatomically complex regions, where thermal injury to adjacent motor fibers, vascular structures, or connective tissue is a concern [50]. However, based on our literature search, no clinical trials of PFA for pain indications have been published. Moreover, electroporation thresholds and dose–response relationships for peripheral nerve tissues relevant to pain management have not been systematically characterized. Accordingly, potential pain indications and therapeutic benefits of PFA remain speculative. The potential of this technology should be understood in the context of these evidence gaps.

## 6. Clinical Translation Across Anatomical Targets

The engineering differences among radiofrequency modalities have direct implications for clinical practice. Modality selection is driven by the anatomy, innervation pattern, and tissue environment of each target, and Table 3 summarizes a suggested mapping of target and clinical goal to modality. The following section illustrates how the biophysical properties of each system map to specific clinical scenarios.

Lumbar Facet Joints. At the lumbar medial branch, conventional thermal monopolar RFA is applied as described in Section 5.1. The medial branch nerve courses predictably along the junction of the transverse and superior articular processes, making it well suited to the elliptical lesion produced by a monopolar electrode placed parallel to the nerve. Responder rates range from 42% to 60% at six months, with pain relief typically lasting 6 to 12 months [1,4,32,44]. The American Society of Pain and Neuroscience endorses conventional thermal ablation as the primary method for facet neurotomy [3]. The success at this target reflects alignment between a predictable nerve course and a reproducible lesion geometry.

Sacroiliac Joint and Sacral Lateral Branches. Sensory innervation of the sacroiliac joint arises from the dorsal ramus of L5 and the lateral branches of S1 through S3, which run in fibro-osseous grooves where fat, ligamentous structures, and bone alter heat conduction unpredictably. The conventional monopolar lesion is often too small to capture these anatomically variable nerves reliably. Cooled RFA produces larger, more spherical lesions (Section 5.2), better matching the variable sacral lateral-branch anatomy [8,30]. Sham-controlled trials demonstrate responder rates of 47–79% with a mean duration of benefit approaching 8 months [39,40]. Multispecialty guidelines support cooled or bipolar ablation at this target because broader lesion fields match the distribution of the sacral lateral branches [46,53].

Basivertebral Nerve. BVN ablation targets nociceptive innervation of the vertebral endplates associated with Modic type I and II changes via a transpedicular approach. The BVN occupies a centrally located intraosseous canal with an arborized terminal nucleus branching toward the superior and inferior endplates, anatomy that demands a lesion wider than a conventional monopolar electrode can produce. Bipolar intraosseous radiofrequency concentrates current density between two closely spaced electrodes within the vertebral body, generating a broad thermal field capable of enveloping the BVN trunk and its branches. Sham-controlled and multicenter randomized trials demonstrate durable reductions in pain and disability for at least five years [42,43,54,55,56,57].

Dorsal Root Ganglion and Radicular Pain. The DRG contains mixed motor and sensory fibers in proximity, making thermal neurodestruction hazardous; PRF is therefore preferred here [10,11,58]. Bipolar pulsed configurations yield superior outcomes compared with monopolar pulsed systems, likely owing to higher interelectrode field intensity, consistent with computational models that show electric-field synergism at appropriate spacings [9,10,11,59]. The principal limitation remains the modest durability of benefit, which has driven adoption of implanted DRG stimulation to sustain symptom control in selected patients.

Peripheral Nerve Pain Syndromes. At peripheral targets, modality selection depends directly on whether motor preservation is required [36,48]. PRF is preferred for mixed or motor-bearing nerves, and thermal ablation for purely sensory nerves, where it yields 6 to 12 months of benefit versus 3 to 6 months for PRF (e.g., thermal ablation of the lateral femoral cutaneous nerve for meralgia paresthetica) [32,36,52].

## 7. Patient-Specific Factors Affecting Lesion Formation

Radiofrequency lesion size and shape are influenced not only by device design and procedural technique but also by the tissue environment. Adipose content, bone proximity, vascular perfusion, and injectables can all alter heating dynamics. An ex vivo model demonstrated that high adipose content reduces lesion size because fat has low thermal conductivity and high impedance; this relationship held across multiple ablation configurations and suggests that radiofrequency lesions in patients with obesity may be smaller than expected for a given set of parameters [60]. The importance of background tissue is further supported by studies describing the so-called oven effect, in which low-thermal-conductivity tissues trap heat, leading to more focal heating [33].

Perfusion also plays a significant role. Highly perfused tissues dissipate heat more quickly, leading to smaller lesions; computational models show that lesion size may vary by more than 100% depending on local blood flow [22]. These findings are especially relevant when performing radiofrequency ablation near vascular structures or in tissues with variable perfusion.

Injectates can also influence lesion geometry. Some evidence suggests that saline or lidocaine can widen lesions, while iodinated contrast may alter conductive properties [61]. Impedance, another key variable, changes dynamically during ablation: high impedance reduces current flow and limits lesion growth, while low impedance allows more aggressive heating. Both ex vivo and clinical studies highlight the importance of real-time impedance monitoring for optimizing lesion predictability [23].

Collectively, these patient-specific factors support the need for individualized procedural planning, particularly for anatomically complex regions or for patients with significant variation in tissue composition. Table 4 compares the lesion geometries produced by conventional, cooled, and bipolar probes, highlighting how probe design and energy delivery influence lesion size and shape, as well as the probability of nerve capture.

## 8. Safety Considerations and Complications

RFA is generally safe, with low rates of serious adverse events across spinal and peripheral applications. The most common effects, including transient soreness, numbness, or neuritis, reflect local inflammation or partial injury to adjacent sensory fibers and usually resolve within days to weeks. Persistent neuropathic pain or motor deficits are rare when proper patient selection and technique are used [1,3,39,62].

Safety also varies by target. DRG and sacroiliac joint procedures require attention to motor stimulation thresholds to avoid unintended motor injury, and more broadly, avoidance of heating near motor fibers, spinal cord structures, or implanted hardware is paramount. BVN ablation has demonstrated no device-related serious adverse events across randomized trials and large cohort studies [42,43,53,54,55,56,57,63]. Sacroiliac joint RFA similarly demonstrates a favorable safety profile in comparative effectiveness trials [39,40,46].

Repeated RFA maintains effectiveness without significant cumulative risk. Studies evaluating repeated lumbar medial branch neurotomy demonstrate consistent clinical benefit, with no evidence of progressive fibrosis that would impair future lesion formation [32]. Cooled and conventional radiofrequency techniques appear to have similar overall safety profiles, as demonstrated in multiple retrospective and prospective analyses [31,64].

Across all targets, anatomically guided placement, sensory and motor stimulation, and careful attention to impedance trajectories are the core measures for minimizing risk.

## 9. Emerging Technologies and Future Directions

The landscape of radiofrequency-based pain management continues to expand as engineers and clinicians develop new methods to shape energy delivery and achieve tissue-selective effects. Several emerging technologies warrant discussion, each at a different stage of preclinical and clinical validation.

Relative Clinical Maturity of Radiofrequency and Energy-Based Modalities. The modalities discussed in this review occupy a spectrum of clinical maturity, ranging from established, guideline-endorsed techniques to investigational and preclinical approaches, and this gradient, rather than any single measure of efficacy, should guide expectations for each technology. Conventional thermal RFA is the most mature: it is the reference standard for lumbar medial branch neurotomy, endorsed by multiple society guidelines and supported by multiple randomized controlled trials [1,3,4]. Cooled and bipolar thermal RFA are established at specific targets, with the strongest evidence at the knee (genicular) and sacroiliac targets, where larger or strip lesions match anatomically variable innervation [39,40,46,47]. Intraosseous bipolar thermal ablation of the basivertebral nerve is RCT-supported, with sham-controlled and multicenter randomized trials demonstrating durable improvement in pain and disability for at least five years [42,43,54]. PRF is widely used but remains contested: although broadly adopted for mixed and motor-bearing nerves, its clinical evidence is heterogeneous and its parameters are not standardized, so its role continues to be debated [32,41]. Hybrid pulsed–thermal systems and high-voltage PRF are investigational, resting on early-phase studies, preclinical data, and systematic-review protocols rather than completed trials with robust outcome data [41,49,65]. Finally, high-intensity focused ultrasound (HIFU) and PFA remain investigational or preclinical for pain indications: HIFU is supported by feasibility and small prospective studies; no clinical trials of PFA for pain have been published, with peripheral-nerve electroporation thresholds still uncharacterized [50,66,67].

Positioning within this spectrum is target-dependent rather than absolute. Cooled, bipolar, and intraosseous thermal techniques are each established or RCT-supported at different anatomical targets, while PRF’s status varies by indication, favored where motor preservation is essential, but limited by its shorter duration of benefit.

Comparison with Other Energy-Based Ablation Modalities. RFA is one of several energy-based technologies used to create therapeutic lesions, and its behavior is best understood in the context of the alternatives (Table 5). Microwave ablation generates heat by dielectric hysteresis, oscillation of water molecules within an electromagnetic field, rather than by ionic conduction. This mechanism enables faster, higher-temperature heating that is less constrained by tissue impedance and less susceptible to the perfusion-mediated heat-sink effect that limits radiofrequency lesions [68,69]. Laser interstitial thermal therapy delivers photothermal energy through a fiberoptic applicator, typically under real-time magnetic resonance thermometry, and is used predominantly for intracranial and spinal tumors rather than for peripheral neural targets [70].

Cryogenic techniques produce the opposite thermal insult, but two mechanistically distinct modalities must be distinguished. Cryoneurolysis applies controlled cold (typically −20 °C to −88 °C, within a broader therapeutic window of approximately −20 °C to −100 °C) to induce a Sunderland grade II axonotmesis, with Wallerian degeneration of the axon distal to the treatment site while the endoneurium, perineurium, and epineurium are preserved; because the connective-tissue scaffolding is retained, the nerve regenerates predictably at roughly 1–2 mm/day, producing temporary, reversible analgesia with a low risk of painful neuroma, an attractive profile for temporary interruption of peripheral neuropathic pain, although the duration of benefit is inherently limited [71].

Cryoablation, by contrast, uses more aggressive freezing (often below −100 °C) to cause coagulative necrosis and permanent, irreversible destruction of the target tissue without connective-tissue preservation or subsequent tissue recovery within the ablated zone. The two therefore differ in intent as well as in degree: cryoneurolysis aims at reversible interruption of nerve conduction through a regenerating grade II injury, whereas cryoablation aims at permanent, irreversible tissue destruction (see Table 5 for temperature ranges and mechanisms).

HIFU achieves noninvasive thermal ablation via focused acoustic energy, whereas PFA achieves nonthermal, tissue-selective ablation via irreversible electroporation (Section 5.6) [50,66,67]. For most neural targets in pain medicine, RFA remains preferred because of its controllability, real-time impedance feedback, modest cost, and extensive clinical validation, while microwave, laser, cryoablation, focused ultrasound, and pulsed-field approaches currently occupy niche or investigational roles [69].

Beyond the modalities detailed in Section 5, several technologies remain at earlier stages of translation. PFA is the most prominent (Section 5.6); its cardiac validation provides a rationale for sparing adjacent motor fibers, vessels, and connective tissue at anatomically complex pain targets [50,51]. Given the absence of clinical pain data, preclinical validation of electroporation thresholds in musculoskeletal and neural models is the necessary next step.

High-voltage PRF is also under investigation, with early protocols suggesting greater neuromodulatory effects than standard pulsed systems [41,65]. Hybrid pulsed–thermal systems (Section 5.5) have shown early promise for conditions such as trigeminal neuralgia, where both targeted neurodestruction and neuromodulation may play therapeutic roles [49]. For both, evidence remains limited to systematic review protocols and early-phase investigations; no completed trials with robust outcome data are yet available, and whether higher-voltage delivery translates into greater or more durable clinical benefit remains to be determined.

HIFU is also gaining attention as a noninvasive ablative modality capable of creating precise thermal lesions within deep musculoskeletal and neural structures without the need for needle insertion. Preclinical swine studies demonstrate the feasibility of fluoroscopy guided HIFU for medial branch nerve ablation, with successful ablation at all targets and no significant adverse events [72,73]. A prospective clinical study (*n* = 30) of fluoroscopy-guided HIFU neurotomy of the lumbar medial branches reported an 82.6% responder rate at six months, comparable to conventional RFA, with no significant device- or procedure-related adverse events [66]; MRI-guided focused ultrasound of painful lumbar facet joints has likewise shown acceptable safety and tolerability in human subjects [74]. Ex vivo and in vivo models show dose-dependent, partly reversible effects on peripheral nerve conduction, in some cases mediated by mechanically gated ion channels such as PIEZO2 [75,76]. Clinical evidence in pain applications remains limited, but these data support HIFU as a promising noninvasive alternative warranting further investigation [67].

Advances in computational modeling continue to refine predictions of lesion geometry and help optimize procedural parameters based on individualized tissue characteristics [22,45]. Machine-learning approaches may enable real-time adjustment of power delivery based on impedance, perfusion, and anatomical feedback.

Together, these innovations point toward a future of highly customizable radiofrequency systems that balance efficacy, safety, tissue selectivity, and adaptability. As preclinical validation matures and clinical trials accumulate, these technologies have the potential to expand the range of treatable conditions and anatomic targets accessible to energy-based pain intervention (Table 1, Table 2, Table 3, Table 4 and Table 5).

## 10. Controversies, Limitations, and Evidence Gaps

Several controversies temper the conclusions as written:

1. Scarcity of head-to-head data. Most evidence derives from single-arm or sham-controlled trials of a single technique, so differences in reported responder rates partly reflect heterogeneous populations, outcome definitions, and follow-up periods rather than true superiority [30,46,64]. Cooled and conventional radiofrequency, for example, have not been shown to differ consistently for lumbar facet pain despite their different lesion geometries, and meta-analyses reach mixed conclusions [30,47,64].
2. Contested role of PRF. Although mechanistic studies demonstrate genuine neuromodulatory effects in preclinical models, clinical trials are heterogeneous, and several reports benefits no greater than, or shorter than, those of thermal ablation, so the clinical role of PRF remains debated [32,41].
3. Reliance on ex vivo and computational lesion data. Much of the lesion-characterization data that guides device selection comes from ex vivo or computational models, and extrapolation to perfused, innervated human tissue is uncertain [8,22,61].
4. Limited independent replication of newer approaches. Durability and patient-selection criteria for newer intraosseous and emerging approaches, BVN ablation, PFA, and focused ultrasound, rest on a limited number of trials, several of which are industry-sponsored, with little long-term independent replication [42,43,56].
5. Narrative-review limitations. As a narrative review, this work prioritizes mechanistic coherence and representative evidence and may underweight negative or unpublished results. These limitations are noted not to diminish the technology but to mark where rigorous, comparative, independently replicated, and adequately powered studies are most needed.

## 11. Conclusions

RFA continues to evolve as a versatile and well-validated tool in interventional pain management. Despite the diversity of available systems, all radiofrequency modalities share a unified biophysical mechanism rooted in ionic agitation and the conversion of alternating electrical current into controlled thermal or electrical effects within biological tissue. A working understanding of the physical principles, tissue interactions, engineering innovations, and clinical evidence allows clinicians to select modalities more rationally, optimize lesion geometry, and tailor procedures to each patient’s anatomical and physiological characteristics.

The growing body of clinical evidence supports RFA as a safe and effective treatment for facet-mediated, sacroiliac joint, vertebrogenic, radicular, and peripheral neuralgic pain. Innovations such as cooled radiofrequency, bipolar configurations, hybrid pulsed–thermal systems, pulsed-field ablation, and BVN ablation continue to broaden its scope, although several, as noted above, still require comparative and long-term validation (Table 1, Table 2, Table 3, Table 4 and Table 5). As device engineering converges with computational modeling and clinical evidence, RFA is likely to remain a central tool in interventional pain management.

## Figures and Tables

**Figure 1:**
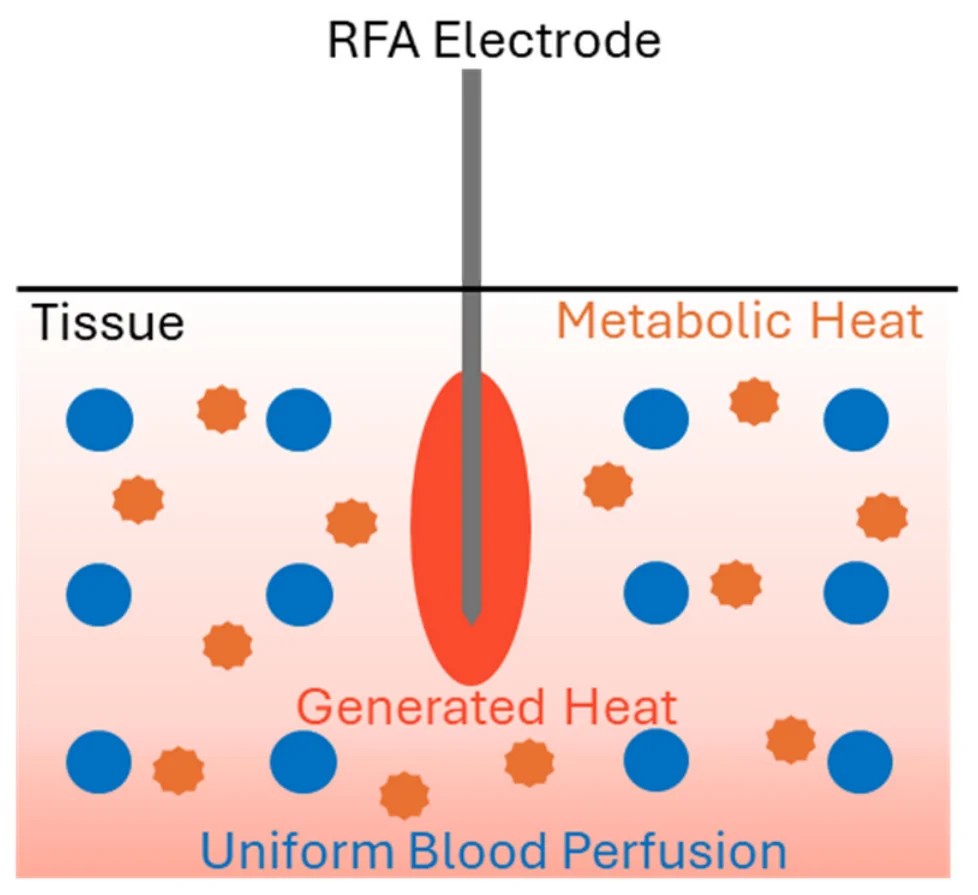
Illustration of the Pennes bioheat model. The vertical grey bar represents the radiofrequency electrode; the red ellipse at the active tip denotes the heat generated by resistive (ionic) heating; the blue circles denote uniformly perfusing blood, which carries heat away from the treated volume; the orange starburst icons denote metabolic heat generation within the tissue; and the background colour gradient indicates the resulting tissue temperature distribution, from higher (red) near the tip to lower (white) at the periphery.

**Figure 2:**
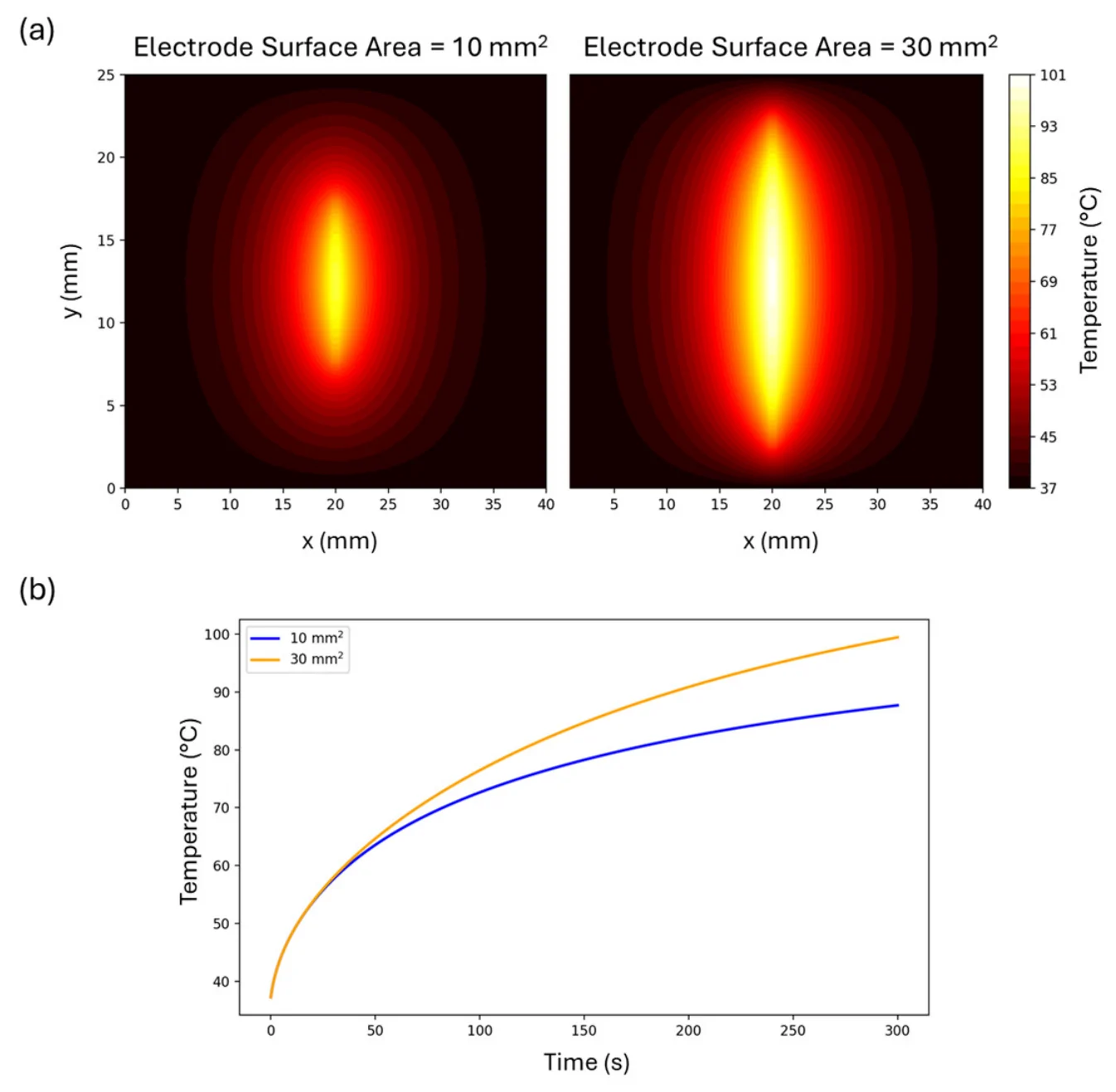
(**a**) Thermal heating gradient for electrodes of two surface areas, 10 mm^2^ (left) and 30 mm^2^ (right). (**b**) Temperature increase versus time at the electrode center for 10 mm^2^ (blue) and 30 mm^2^ (orange) electrodes.

**Figure 3:**
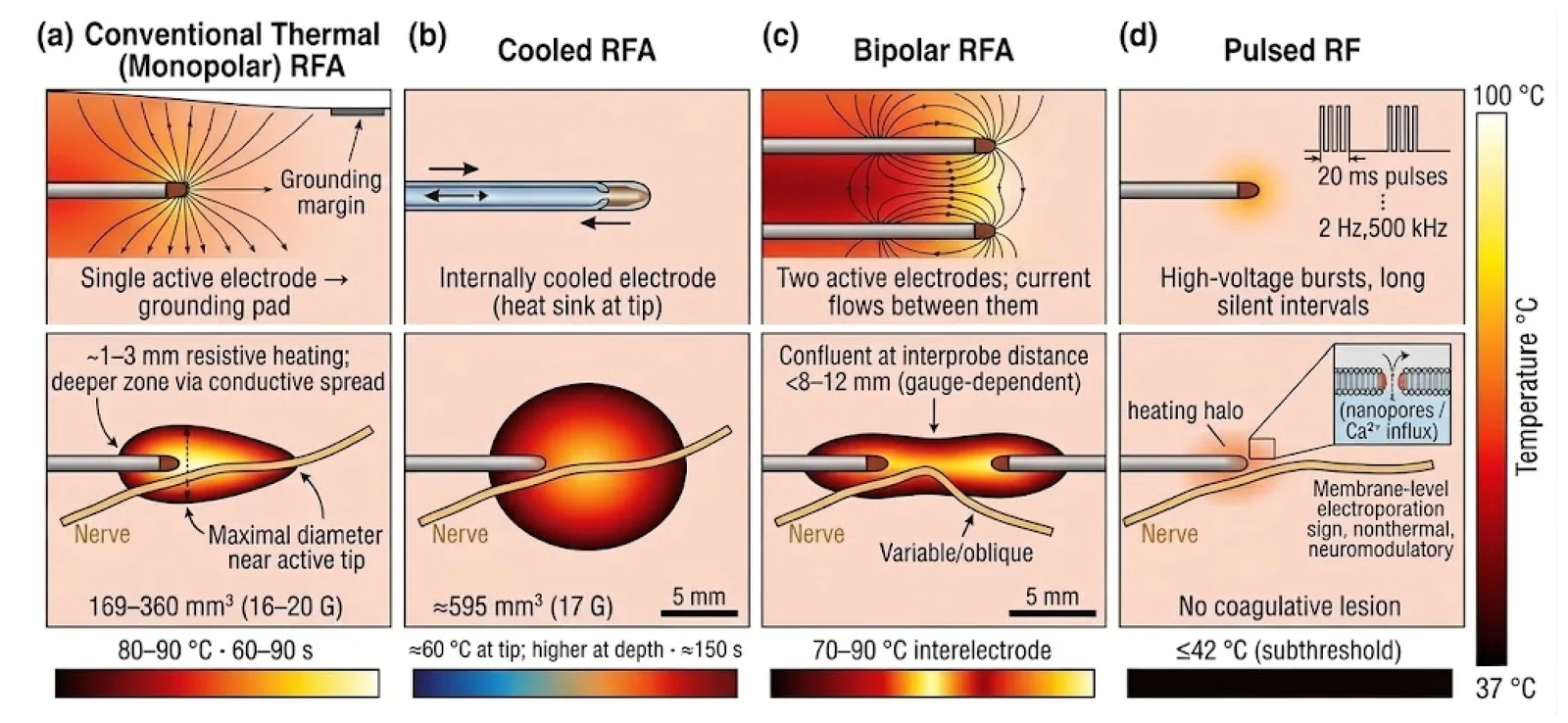
Electrode configuration, lesion geometry, and thermal behavior across the four principal radiofrequency modalities. (**a**) Conventional monopolar thermal RFA produces an elliptical, impedance-limited lesion at 80–90 °C. (**b**) Internally cooled RFA prevents surface charring, permitting higher power deposition and a larger, more spherical lesion. (**c**) Bipolar RFA concentrates current between two active electrodes, generating a confluent strip lesion when interprobe distance is below the gauge-dependent confluence threshold. (**d**) Pulsed RF delivers subthreshold (≤42 °C) high-voltage bursts that produce neuromodulation via electric-field/electroporation effects without a coagulative lesion. Arrows denote the direction of current flow from the active electrode to the dispersive (grounding) pad in (**a**) and between the two active electrodes in (**c**), the direction of circulating internal perfusate in (**b**), and the pulse train and inter-pulse interval in (**d**); leader arrows elsewhere identify the lesion feature named by the adjacent label. Temperature scale and quantitative parameters correspond to Table 1 and Table 2.

**Table 1:** Mechanistic distinctions among RFA modalities.

RFA Modality	Primary Mechanism of Action	Temperature Profile	Electrical Field Characteristics	Biological Effect on Nerve	Clinical Rationale
Conventional Thermal RFA	Heat generation through ionic agitation and friction in tissue surrounding the active electrode	Maintains 80–90 °C for coagulative necrosis	Current density highest at electrode tip, decays steeply with distance	Irreversible axonal injury and Wallerian degeneration	Ideal for small, predictable sensory nerves such as lumbar medial branches
Cooled RFA	Same thermal mechanism as conventional RFA, with enhanced power delivery due to internal cooling	Maintains 60 °C at electrode surface while allowing higher internal tissue temperatures	Cooling prevents desiccation and impedance rise, permitting deeper current penetration	Larger, more spherical lesions increase probability of nerve capture	Suited for anatomically variable targets such as sacral lateral branches
Bipolar Thermal RFA	Concentrated electric field between two active electrodes producing an elongated thermal corridor	Interelectrode temperatures reach 70–90 °C depending on parameters	Field convergence enhances heating in the tissue between probes	Continuous strip lesion with consistent neurodestruction	Useful for DRG, sacroiliac complex, and applications requiring extended lesion length
Pulsed Radiofrequency	Nonthermal modulation through high-voltage bursts and electric field-induced membrane effects	Keeps tissue below 42 °C to avoid coagulative necrosis	Strong but intermittent electric fields that alter membrane potentials	Neuromodulation through altered axonal transport, reduced neuropeptides, and transient electroporation (proposed; largely preclinical)	Preferred for mixed nerves or motor-sparing applications such as occipital or peripheral neuralgias
Hybrid Pulsed–Thermal Systems	Alternating cycles of pulsed and mild thermal energy	Moderately elevated temperatures (45–60 °C) depending on device	Combined electric and thermal effects	Partial neurodestruction with enhanced neuromodulation (proposed; largely preclinical)	Investigational use for trigeminal neuralgia and chronic neuropathic pain
Pulsed Field Ablation (Irreversible Electroporation)	Cell death induced by high-voltage, ultrashort electrical pulses without relying on thermal injury	Temperature remains near physiologic levels	Nonthermal electroporation produces tissue-selective disruption	Targets neuronal membranes while sparing connective tissues (proposed; largely preclinical)	Emerging technology with potential safety advantages for complex anatomy

**Table 2:** Representative quantitative parameters and clinical outcomes by radiofrequency modality. Values are drawn from separate ex vivo and clinical studies that differ in patient population, treatment target, stimulation protocol, outcome definition, and follow-up interval. Entries are therefore not directly comparable and should not be read as head-to-head results; responder rates and durations of benefit are reported only to characterize each modality within its own evidence base.

Modality	Temperature/Settings	Representative Lesion Size	Duration of Benefit	Responder Rate (≥50% Relief)	Evidence Base
Conventional thermal (monopolar)	80–90 °C, 60–90 s	169–360 mm^3^ (16–20 G) [8]	6–12 months	42–60% (lumbar facet, 6 mo) [1,4]	Multiple RCTs and guidelines [1,3,4]
Cooled	≈60 °C tip, ≈150 s	≈595 mm^3^ (17 G) [8]	≈8 months (sacroiliac) [39]	47–79% (sacroiliac) [39]	Sham-controlled RCTs [40]
Bipolar	70–90 °C interelectrode	Confluent strip; confluence at IPD <8–12 mm [7]	Comparable to thermal	Reported benefit in DRG/radicular pain; comparative data limited [10,11]	Ex vivo and RCT data [7,10,11]
Pulsed	≤42 °C; 20 ms at 2 Hz, 120 s	No coagulative lesion	3–6 months	Variable; target-dependent [32,41]	Heterogeneous RCTs and reviews [32,41]
Bipolar intraosseous (basivertebral nerve)	Intraosseous bipolar thermal	Envelops arborized BVN within vertebral body	≥5 years	Durable ODI/VAS improvement [42,43]	Sham-controlled and multicenter RCTs [43,44]

**Table 3:** Suggested radiofrequency modality selection by neural target and clinical goal. This table presents a mechanistically reasoned mapping, not a ranking derived from comparative trials. The listed durations and relief estimates originate from heterogeneous single-modality studies and are provided for orientation only, not as evidence of superiority of one modality over another at a given target.

Target/Scenario	Primary Goal	Key Tissue or Anatomic Factor	Preferred Modality	Biophysical Rationale
Lumbar facet (medial branch)	Neurodestruction	Predictable nerve along bone	Conventional thermal monopolar	Reproducible ellipsoidal lesion aligned to the nerve [1,4]
Sacroiliac lateral branches (S1–S3)	Neurodestruction over variable anatomy	Variable course in fibro-osseous grooves	Cooled or bipolar	Larger or strip lesions raise capture probability [39,40,45]
Basivertebral nerve	Intraosseous neurodestruction	Central arborized canal within bone	Bipolar intraosseous	Confluent intraosseous field envelops the arbor [42,43]
Dorsal root ganglion/radicular	Neuromodulation (motor-sparing)	Mixed motor and sensory fibers	Pulsed (bipolar pulsed)	Sub-ablative field modulates without motor injury [10,11]
Peripheral mixed or motor nerve	Neuromodulation	Motor fibers present	Pulsed	Preserves neural structure [41,48]
Peripheral sensory nerve	Neurodestruction (longer relief)	Purely sensory	Thermal (or pulsed)	Safe to ablate; 6–12 months of benefit [32,52]

**Table 4:** Comparison of lesion geometry across conventional, cooled, and bipolar RFA probes.

Probe Type	Typical Lesion Shape	Determinants of Lesion Size	Advantages	Limitations	Representative Clinical Use
Conventional Thermal Probe	Elliptical or teardrop shaped, narrowest at distal end	Current density, tissue impedance, rapid desiccation that limits expansion	Simple predictable lesion for parallel nerve contact	Limited lesion volume due to impedance rise, sensitive to tissue variability	Lumbar medial branch neurotomy
Cooled RFA Probe	Spherical or ovoid with significantly greater volume than conventional probes	Internal cooling maintains conductivity, allows high-power deposition without charring	Larger uniform lesions that compensate for anatomic variability	Requires specialized equipment and larger introducers	Sacroiliac joint lateral branch ablation, BVN ablation
Bipolar RFA Probes	Elongated strip lesion bridging the electrodes	Interprobe distance, gauge, and field convergence; optimal spacing < 10–12 mm	Creates continuous lesion across broader neural territory	Loss of confluence if spacing is excessive; more complex positioning	Dorsal root ganglion treatments, sacral base neurotomy
Pulsed Radiofrequency Probe	No true thermal lesion, only a small heating halo	Electric field strength and pulse configuration	Minimizes structural damage for motor-sparing targets	Shorter duration of pain relief	Occipital and peripheral nerve neuromodulation

**Table 5:** Comparison of RFA with other energy-based ablation modalities used or investigated in pain management.

Modality	Energy Source and Primary Mechanism	Thermal vs. Nonthermal	Characteristic Lesion	Role in Pain Medicine
Conventional/Cooled RFA	High-frequency AC; ionic friction (resistive heating)	Thermal (60–90 °C)	Ellipsoidal; cooled probes give larger, more spherical lesions; impedance-limited	Reference standard for facet, sacroiliac, genicular, and vertebrogenic targets
Pulsed Radiofrequency	High-voltage bursts; electric-field effects and reversible electroporation	Nonthermal (≤42 °C)	No coagulative lesion; small heating halo	Mixed or motor-bearing nerves (DRG, occipital, peripheral); shorter benefit
Microwave ablation	Electromagnetic field; dielectric hysteresis (water-molecule oscillation)	Thermal (often >100 °C)	Large, rapid; less impedance- and perfusion-limited than RFA	Oncologic and osseous; limited neural-pain data
Laser (LITT)	Photothermal absorption via fiberoptic applicator; MR-thermometry guided	Thermal	Small, precisely controlled coagulation	Chiefly intracranial and spinal tumors; rare for peripheral pain
Cryoneurolysis *	Compressed nitrous oxide (N_2_O) or carbon dioxide (CO_2_) generates localized Joule–Thomson cooling (therapeutic window approximately −20 °C to −100 °C; practical floor set by the gas boiling point, ≈−88 °C for N_2_O and ≈−79 °C for CO_2_), inducing a Sunderland grade II axonotmesis with Wallerian degeneration of the axon distal to the treatment site while the endoneurium, perineurium, and epineurium are preserved	Thermal (cold-induced)	Reversible Sunderland grade II nerve injury with intact connective tissue scaffolding, allowing predictable axonal regeneration (~1–2 mm/day) over weeks to months	Temporary, reversible analgesia in peripheral nerve-mediated pain (e.g., occipital neuralgia, post-thoracotomy pain, knee osteoarthritis, post-amputation pain, intercostal neuralgia) when long-lasting but reversible nerve blockade is desired
Cryoablation *	Argon-based cryoprobes generate rapid freezing (often <−100 °C), frequently combined with helium-mediated thaw cycles, producing intracellular ice crystal formation, osmotic injury, microvascular thrombosis, and coagulative necrosis of the target tissue	Thermal (cold-induced)	Well-demarcated zone of coagulative necrosis with permanent, irreversible destruction of the intended target tissue (e.g., tumor, ganglion, or selected nerve).	Primarily used for oncologic ablation (bone, liver, kidney, lung, prostate tumors) and selected pain interventions requiring permanent destruction of painful lesions or targeted neural structures
High Intensity Focused Ultrasound	Focused acoustic energy; mechanical and thermal heating	Thermal (noninvasive)	Precise deep focal lesion, no needle insertion	Investigational for facet/medial-branch and peripheral nerve targets
Pulsed-field ablation	High-voltage, ultrashort pulses; irreversible electroporation	Nonthermal	Tissue-selective, wider, and largely perfusion-independent	Established in cardiac electrophysiology; investigational for pain

* Cold is a thermal insult; cryomodalities are classified as thermal by mechanism.

## Data Availability

No new data were created or analyzed in this study. Data sharing does not apply to this article.

## Abbreviations

The following abbreviations are used in this manuscript:

RFA	Radiofrequency ablation
BVN	Basivertebral nerve
PRF	Pulsed radiofrequency
DRG	Dorsal root ganglion
PFA	Pulsed-field ablation
HIFU	High-intensity focused ultrasound
IPD	Interprobe distance
CGRP	Calcitonin gene-related peptide
AC	Alternating current
